# Association of Primary Intracerebral Hemorrhage With Pregnancy and the Postpartum Period

**DOI:** 10.1001/jamanetworkopen.2020.2769

**Published:** 2020-04-14

**Authors:** Jennifer R. Meeks, Arvind B. Bambhroliya, Katie M. Alex, Sunil A. Sheth, Sean I. Savitz, Eliza C. Miller, Louise D. McCullough, Farhaan S. Vahidy

**Affiliations:** 1 Center for Outcomes Research, Houston Methodist Research Institute, Houston, Texas; 2 Department of Neurology, McGovern Medical School, UTHealth, Houston, Texas; 3 Department of Neurology, Columbia University Vagelos College of Physicians and Surgeons, New York, New York

## Abstract

**Importance:**

Intracerebral hemorrhage (ICH) during pregnancy and the postpartum period results in catastrophic maternal outcomes. There is a paucity of population-based estimates of pregnancy-related ICH risk, including risk during the extended postpartum period.

**Objective:**

To evaluate ICH risk during pregnancy and an extended 24-week postpartum period in a population-level cohort and to determine fetal and maternal outcomes as well as demographic and comorbidity factors associated with ICH during pregnancy and post partum.

**Design, Setting, and Participants:**

This study used a cohort-crossover design in which patients serve as their own controls when no longer exposed (pregnant or post partum). Administrative data were obtained from all hospital admissions for New York, California, and Florida for a 7- to 10-year period. Participants included all women admitted for labor and delivery who were older than 12 years and did not have a prior diagnosis of ICH. Conditional Poisson regression models were used to evaluate ICH risk, and data were reported as rate ratios and 95% CIs. Data analysis was performed from August 2018 to February 2020.

**Exposures:**

Women were tracked using hospitalization records for the duration of pregnancy (40 weeks), for 24 weeks post partum, and for an additional 64 weeks when no longer exposed.

**Main Outcomes and Measures:**

Diagnosis of ICH during both 64-week observation periods was determined using validated *International Classification of Diseases, Ninth Revision* codes.

**Results:**

A total of 3 314 945 pregnant women were included (mean [SD] age, 28.17 [6.47] years; 1 451 780 white [43.79%], 474 808 black [14.32%], 246 789 Asian [7.44%], and 835 917 Hispanic [25.22%]). The risk of ICH was significantly higher during the third trimester (2.9 vs 0.7 cases per 100 000 pregnancies; rate ratio, 4.16; 95% CI, 2.52-6.86) and remained elevated during the first 12 weeks post partum (4.4 vs 0.5 cases per 100 000 pregnancies; rate ratio, 9.15; 95% CI, 5.16-16.23). Advanced maternal age (adjusted odds ratio [OR], 1.08; 95% CI, 1.05-1.10), nonwhite race (adjusted ORs, 2.44 [95% CI, 1.73-3.44] for black patients, 2.12 [95% CI, 1.34-3.35] for Asian patients, and 1.59 [95% CI, 1.12-2.26] for Hispanic patients), hypertension (adjusted OR, 2.02; 95% CI, 1.19-3.42), coagulopathy (adjusted OR, 14.17; 95% CI, 9.17-21.89), preeclampsia or eclampsia (adjusted OR, 9.23; 95% CI, 6.99-12.19), and tobacco use (adjusted OR, 2.83; 95% CI, 1.53-5.23) were independently associated with ICH during pregnancy and the postpartum period. Pregnancy-related ICH was associated with a higher risk of maternal (relative risk difference, 792.6; absolute risk difference, 0.18) and fetal (relative risk difference, 5.3; absolute risk difference, 0.03) death, compared with pregnancies without ICH.

**Conclusions and Relevance:**

These findings suggest that the risk of ICH is significantly higher during the third trimester of pregnancy and the first 12 weeks post partum. There are age and race disparities in ICH risk that are associated with devastating maternal and fetal outcomes. These data illustrate the critical need for continuous monitoring and aggressive management of ICH-associated risk factors. These findings suggest that extended postpartum monitoring of high-risk women may be warranted.

## Key Points

**Question:**

What is the population-level risk of intracerebral hemorrhage during pregnancy and an extended postpartum period, and what is the association between this risk and maternal and fetal mortality?

**Findings:**

This cohort-crossover study of 3 314 945 pregnancies found an increased rate of intracerebral hemorrhage during the third trimester (2.9 vs 0.7 cases per 100 000 pregnancies) and the first 12 postpartum weeks (4.4 vs 0.5 cases per 100 000 pregnancies). Maternal and fetal mortality were higher among women who experienced intracerebral hemorrhage, and age and race disparities were also observed.

**Meaning:**

These findings suggest that the increased risk of intracerebral hemorrhage into 12 weeks post partum warrants extended postnatal monitoring of high-risk women.

## Teaser

This cohort-crossover study evaluates the risk of intracerebral hemorrhage during pregnancy and an extended 24-week postpartum period and examines the fetal and maternal outcomes, as well as associated demographic and comorbidity factors.

## Introduction

Sex-specific risk factors, including pregnancy, are increasingly recognized to play an important role in the incidence, causes, severity, hemorrhage characteristics, and outcomes among patients with nontraumatic intracerebral hemorrhage (ICH).^1,2,3,4^ However, studies^5^ quantifying the risk of ICH during pregnancy and the postpartum period are limited. Challenges in assessing the risk of a rare outcome, along with the need to control for several confounders, limit the interpretational value of findings from small, single-center, cross-sectional studies. Furthermore, to our knowledge, the risk of ICH in an extended postpartum period has not been evaluated, even though a higher risk of thrombotic events has been reported up to 12 weeks after pregnancy.^6^

We sought to determine the risk of ICH during pregnancy and an extended 24-week postpartum period using data from statewide hospitalizations in New York, Florida, and California across 7 to 10 years. We used a cohort-crossover design in which pregnant and postpartum women serve as their own controls when no longer pregnant or during the postpartum period. We further explored factors associated with ICH during pregnancy and the postpartum period and describe maternal and fetal outcomes among women who experience ICH during pregnancy.

## Methods

### Data Source

We analyzed data from the Agency for Healthcare Research and Quality Healthcare Cost and Utilization Project’s State Inpatient Database and State Emergency Department Database. Along with a core set of demographic and comorbidity variables, the State Inpatient Database and State Emergency Department Database include diagnostic and procedure codes for all discharges from community hospitals in the respective state for all insurance types and for uninsured patients. The revisit variable in the database is a unique linkage variable allowing longitudinal tracking of patients. We selected New York (2005-2014), California (2005-2011), and Florida (2005-2014) databases on the basis of the availability of linkage information across long periods. Furthermore, this selection allowed us to generate an ethnically and geographically diverse sample accounting for approximately 25% of the US population.

The study investigators completed training and signed a data use agreement. The use of publicly available deidentified data did not warrant an institutional board review or the need for informed consent for this study, in accordance with 45 CFR §46. This study follows the Strengthening the Reporting of Observational Studies in Epidemiology (STROBE) reporting guideline.

### Study Design

**Figure 1.**
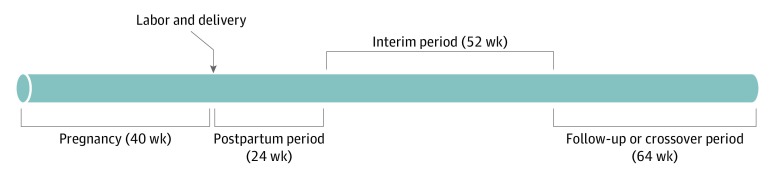
Cohort-Crossover Design With Respective Observation and Interim Periods Patients were identified at the time of labor and delivery (denoted by the arrow) and were retrospectively observed for the 40 weeks of pregnancy and prospectively observed for a 24-week postpartum period, resulting a 64-week cohort period. At the end of the cohort period, patients were observed for a 52-week interim period for death and subsequent pregnancy. At the end of the 52-week interim period, at-risk patients (alive and not pregnant or in a postpartum period) were again observed for a 64-week crossover period.

We used a cohort-crossover design in which patients served as their own controls. To implement this method, we identified a cohort of eligible women with labor and delivery events. The cohort was then observed for diagnosis of ICH over a 64-week cohort period, starting 40 weeks before the labor and delivery event (pregnancy) and extending 24 weeks after the labor and delivery event (post partum). At the close of the cohort period, the individuals were followed for a 52-week interim period for death and subsequent pregnancies. At the end of the interim period, at-risk (alive) women were again observed for a 64-week crossover period (Figure 1). Each patient was followed for a total of 180 weeks. Those who were pregnant or postpartum during the crossover period were not included in at-risk women and were excluded from the follow-up cohort.

### Patient Population, Exposure, and Outcomes

**Figure 2.**
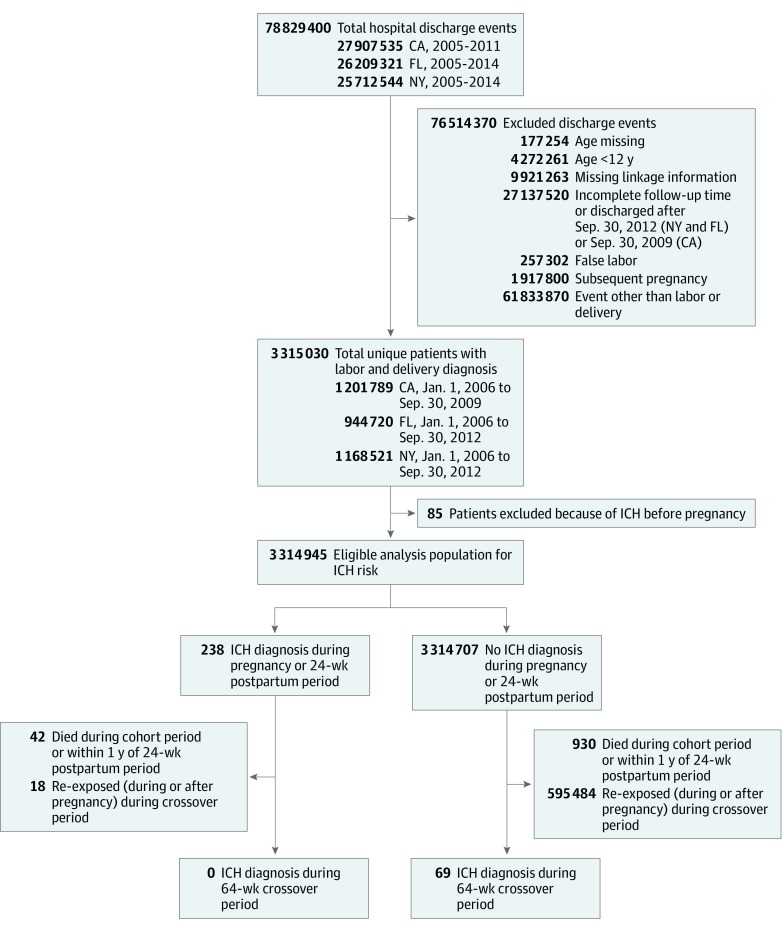
Diagram Indicating Eligible, Excluded, and Analysis Population Beginning With Total Hospital Discharges for New York (NY), California (CA), and Florida (FL) for the Duration of the Observation Period Patients younger than age 12 years, missing linkage information, admitted outside of the pregnancy observation time window, and those with false labor, subsequent pregnancies, and nonlabor or delivery admissions were excluded. The exclusion criteria are not mutually exclusive, and patients may fall into more than 1 exclusion category. More than 3 million unique patients with labor and delivery diagnosis were included. Among these patients, those with a prior diagnosis of intracerebral hemorrhage (ICH) were also excluded, resulting in the eligible analysis population for ICH risk. The eligible population was divided into those who did and did not have an ICH diagnosis during pregnancy and the postpartum period. Those who died before the 64-week crossover period and those who were reexposed (pregnant or postpartum period) during the crossover period were excluded from the final matched cohort. Jan indicates January; and Sep, September.

We identified all labor and delivery events using previously reported *International Classification of Diseases, Ninth Revision *(*ICD-9*) codes related to vaginal or cesarean delivery (72-75, V27, and 650-659)^6,7^ between January 1, 2006, and September 30, 2009, for California; January 1, 2006, and September 30, 2012, for Florida; and January 1, 2006, and September 30, 2012, for New York to ensure that all patients were followed up for the full duration of the follow-up period. We excluded patients younger than age 12 years, those with missing linkage information, and those with a diagnosis of ICH before pregnancy for the duration of available data, at minimum 1 year. For patients with multiple pregnancies, we included only the first pregnancy event. Individuals with multiple labor or delivery events within a 40-week period were considered to have experienced false labor and only the final labor- and delivery-related hospital admission was included (Figure 2). Nontraumatic ICH was identified using the validated *ICD-9* code (431) with 85% sensitivity,^8^ 96% specificity,^8^ and 89% to 97% positive predictive value^8,9,10^ in any diagnosis position. Those with concurrent diagnoses of ICH and head trauma, defined using *ICD-9* codes recommended by the Centers for Disease Control and Prevention for identification of traumatic brain injury, were not included in analysis.^11,12^ We further determined fetal mortality at the delivery event and maternal mortality within 1 year and conducted exploratory analyses of potential ICH causes, including arteriovenous malformation, cerebral venous sinus thrombosis, and sickle cell anemia, using associated diagnosis and procedure codes.

### Statistical Analysis

We stratified the cohort period into the 3 trimesters of pregnancy and 2 12-week postpartum periods. We used conditional Poisson regression to compare the incidence of ICH in our matched population and reported risk differences and rate ratios (RRs) along with 95% CIs. We evaluated the association between various demographic characteristics and comorbidities and ICH during pregnancy and the postpartum period using logistic regression, retaining only statistically significant variables (defined as *P* < .05) in our final adjusted model. We provide summary statistics and both unadjusted and adjusted estimates as odds ratios (ORs) and 95% CIs. All analyses were conducted between August 2018 and February 2020 using Stata statistical software version 15 (StataCorp).

## Results

### Cohort Characteristics

A total of 3 314 945 pregnant women were included in our cohort, with a mean (SD) age of 28.17 (6.47) years. There were 1 451 780 white (43.79%), 474 808 black (14.32%), 246 789 Asian (7.44%), and 835 917 Hispanic (25.22%) women. For this cohort, fewer than 5% of women were uninsured (Table 1).

**Table 1.** Demographic Characteristics, Risk Factors, and Comorbidities of the Patient Population Overall, With and Without ICH

Variable	Participants, No. (%)		
	Overall (N = 3 314 945)	With ICH (n = 238)	Without ICH (n = 3 314 707)
Demographic characteristics			
Age, mean (SD), y	28.17 (6.47)	31.39 (7.10)	28.17 (6.46)
Race^a^			
White	1 451 780 (43.79)	74 (31.09)	1 451 706 (43.8)
Black	474 808 (14.32)	63 (26.47)	474 745 (14.32)
Hispanic	835 917 (25.22)	57 (23.95)	835 860 (25.22)
Asian	246 789 (7.44)	25 (10.50)	246 761 (7.44)
With health insurance^b^	3 210 014 (96.84)	229 (96.23)	3 209 761 (96.84)
Medical history, comorbidities, and risk factors			
Hypertension	52 546 (1.59)	17 (7.14)	52 529 (1.58)
Gestational hypertension	142 405 (4.30)	39 (16.39)	142 365 (4.29)
Diabetes	238 142 (7.18)	36 (15.13)	238 103 (7.18)
Eclampsia or preeclampsia	59 319 (4.32)	84 (35.29)	143 235 (4.32)
Multiple gestation	59 319 (1.79)	NR^c^	NR (1.79)^d^
Coagulopathy	13 437 (0.41)	24 (10.08)	13 413 (0.40)
Thrombocytopenia	29 941 (0.90)	12 (5.04)	29 928 (0.90)
Cocaine use	11 096 (0.33)	NR^c^	NR (0.33)^d^
Tobacco use	65 583 (1.98)	11 (4.62)	65 568 (1.98)
Alcohol use	9680 (0.29)	NR^c^	NR (0.29)^d^

Abbreviations: ICH, intracerebral hemorrhage; NR, not reportable.

^a^ Information regarding race was missing for 2.99% of the overall cohort.

^b^ Health insurance status was missing for less than 0.01% of the overall cohort.

^c^ Number and percentage of patients not reportable because of small number of patients with ICH as dictated by Healthcare Cost and Utilization Project data use agreement.

^d^ Number of patients not reportable because of small number as dictated by Healthcare Cost and Utilization Project data use agreement.

### Risk of ICH During Pregnancy and the Postpartum Period

**Figure 3.**
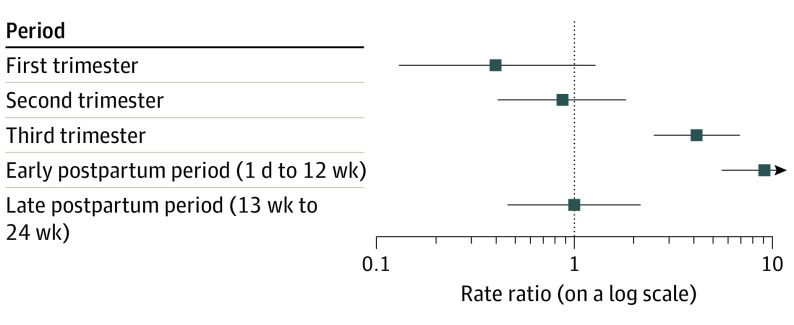
Rate Ratios for Intracerebral Hemorrhage During Pregnancy and Post Partum as Determined by Conditional Poisson Regression in a Matched Patient Population The 64-week matched observation period of 2 719 443 patients is stratified into the 3 trimesters of pregnancy and 2 12-week postpartum periods. Rate ratios are indicated by squares and associated 95% confidence intervals are indicated by horizontal error bars. A dashed vertical line is present at 1 as a reference line for statistical significance.

During the 64-week cohort period, 238 women were considered to have a diagnosis of ICH on the basis of the *ICD-9* code, resulting in an incidence of 7.18 cases per 100 000 pregnancies. In comparison, among the subsequent 2 719 433 at-risk women during the 64-week crossover period, 68 experienced ICH, resulting in an incidence of 2.5 cases per 100 000 individuals. On the basis of our analyses of the matched patient population, excluding those reexposed (had pregnancy or labor event) during the crossover period, the risk of ICH was significantly associated with pregnancy or the postpartum period (risk difference, 5.59; 95% CI, 4.37-6.81; RR, 3.24; 95% CI, 2.46-4.25). The risk of ICH was higher significantly during the third trimester (2.9 vs 0.7 cases per 100 000 pregnancies; RR, 4.16; 95% CI, 2.52-6.86) and first 12-week postpartum period (4.4 vs 0.5 cases per 100 000 pregnancies; RR, 9.15; 95% CI, 5.16-16.23) (Figure 3).

### Factors Associated With ICH During Pregnancy and the Postpartum Period

Women who experienced ICH during pregnancy or the postpartum period were more likely to be older (unadjusted OR, 1.07; 95% CI, 1.05-1.09) and were more likely to be black (unadjusted OR, 2.18; 95% CI, 1.59-3.00) or Asian (unadjusted OR, 1.68; 95% CI, 1.09-2.60) compared with white women. Certain comorbidities, including preeclampsia or eclampsia (unadjusted OR, 10.82; 95% CI, 8.36-14), hypertension (unadjusted OR, 4.31; 95% CI, 2.64-7.05), and coagulopathy (unadjusted OR, 24.82; 95% CI, 16.31-37.78), were also significantly associated with ICH during pregnancy and the postpartum period (Table 2). After adjusting for multiple comorbidities and demographic factors, age (adjusted OR, 1.08; 95% CI, 1.05-1.10), nonwhite race (adjusted ORs, 2.44 [95% CI, 1.73-3.44] for black patients, 2.12 [95% CI, 1.34-3.35] for Asian patients, and 1.59 [95% CI, 1.12-2.26] for Hispanic patients), hypertension (adjusted OR, 2.02; 95% CI, 1.19-3.42), gestational hypertension (adjusted OR, 2.73; 95% CI, 1.91-3.91), eclampsia or preeclampsia (adjusted OR, 9.23; 95% CI, 6.99-12.19), coagulopathy (adjusted OR, 14.17; 95% CI, 9.17-21.89), and tobacco use (adjusted OR, 2.83; 95% CI, 1.53-5.23) were independently associated with ICH during pregnancy or the postpartum period (Table 2).

**Table 2.** Unadjusted and Adjusted ORs for Association Between Intracerebral Hemorrhage and Various Demographic Characteristics, Risk Factors, and Comorbidities

Variable	OR (95% CI)	
	Unadjusted	Adjusted
Demographic characteristics		
Age	1.07 (1.05-1.09)	1.08 (1.05-1.10)
Race		
White	1 [Reference]	1 [Reference]
Black	2.18 (1.59-3)	2.44 (1.73-3.44)
Hispanic	1.13 (0.81-1.56)	1.59 (1.12-2.26)
Asian	1.68 (1.09-2.6)	2.12 (1.34-3.35)
With health insurance	0.92 (0.47-1.78)	NA^a^
Medical history, comorbidities, and risk factors		
Hypertension	4.31 (2.64-7.05)	2.02 (1.19-3.42)
Gestational hypertension	4.01 (2.87-5.62)	2.73 (1.91-3.91)
Diabetes	2.26 (1.61-3.18)	NA^a^
Eclampsia or preeclampsia	10.82 (8.36-14)	9.23 (6.99-12.19)
Multiple gestation	1.95 (1.00-3.8)	NA^a^
Coagulopathy	24.82 (16.31-37.78)	14.17 (9.17-21.89)
Thrombocytopenia	5.73 (3.28-10.01)	NA^a^
Cocaine use	2.52 (1.63-10.15)	NA^a^
Tobacco use	2.40 (1.31-4.40)	2.83 (1.53-5.23)
Alcohol use	2.89 (0.72-11.64)	NA^a^

Abbreviations: NA, not applicable; OR, odds ratio.

^a^ Not included in adjusted model.

In our cohort, approximately one-third (35.29%) of women who experienced ICH had eclampsia or preeclampsia. Also, 9.20% of patients with ICH also had a diagnosis of nonspecific coagulopathy, among whom one-half had disseminated intravascular coagulation. In both the cohort and crossover periods, the number of patients diagnosed with ICH and arteriovenous malformation was fewer than 10. Similarly, we identified fewer than 10 patients with cerebral venous sinus thrombosis and no cases of sickle cell anemia in our analysis cohort. In addition, patients with ICH were also more likely to undergo cesarean delivery compared with patients without an ICH (unadjusted OR, 2.81; 95% CI, 2.17-3.64).

We also conducted 2 sensitivity analyses. In the first, women were observed for ICH 64 weeks before pregnancy, which also resulted in a significantly increased risk of ICH during the third trimester (RR, 8.00; 95% CI, 4.00-16.00) and the first 12 weeks of the postpartum period (RR, 16.00; 95% CI, 7.46-34.34). In the second analysis, we excluded women without a hospital admission or emergency department visit after the 52-week interim period to account for the potentiality of relocating out of state. This analysis continued to demonstrate an elevated ICH risk during the extended (12-week) postpartum period (RR, 4.38; 95% CI, 2.40-8.01).

### Maternal and Fetal Outcomes

Of the 238 women who experienced an ICH during pregnancy or the postpartum period, 42 (17.65%) died. Among these, 20 died during the inpatient stay associated with the delivery event. This finding suggests that maternal mortality associated with ICH during pregnancy and the postpartum period is high (OR, 84.69; 95% CI, 53.59-133.84; adjusted for age and Charlson Comorbidity Index score). Fetal death was also observed in 3.36% of ICH cases during pregnancy vs 0.64% of non-ICH cases (unadjusted OR, 5.40; 95% CI, 2.67-10.92). Pregnancy-related ICH was associated with a higher risk of maternal (relative risk difference, 792.6; absolute risk difference, 0.18) and fetal (relative risk difference, 5.3; absolute risk difference, 0.03) death, compared with pregnancies without ICH.

## Discussion

This study found that the risk of nontraumatic ICH is increased during pregnancy and the postpartum period. The elevated risk of ICH was highest during the third trimester and the first 12 weeks of the postpartum period. To our knowledge, this is the first population-based study to use a cohort-crossover design to evaluate ICH risk in pregnancy and an extended postpartum period and also the first to evaluate fetal outcomes associated with maternal ICH at the population level. The strengths of our study include the use of a large data source that provides estimates for a rare event in a widely generalizable population that includes racial minorities. This study also incorporates design elements that minimize confounding by determining ICH risk in the same cohort of women across 2 distinct time periods.

On the basis of our analyses, we estimate the incidence of nontraumatic ICH during pregnancy and postpartum as 7.18 cases per 100 000 deliveries. Higher estimates (12.2 cases per 100 000 deliveries) have been reported previously when other intracranial hemorrhage types (eg, subarachnoid hemorrhage) were included.^13^ Conversely, our estimates, which are based on analyses of contemporary time periods, are higher compared with those found in earlier cross-sectional reports (6.1 cases per 100 000 deliveries).^7^ An earlier investigation^14^ using data from 1988 and 1991 for 2 US metropolitan areas provides evidence of higher ICH risk during pregnancy and the early postpartum period, albeit without specifying the risk differential between various trimesters. Similar evidence of elevated ICH risk in the early postpartum period also exists for non-US patient populations.^15^ Although our findings broadly confirm these prior reports and other reviews,^13,16^ there are differences in risk estimates. Temporal trends in risk profiles, differences in population characteristics, design effects (cross-sectional vs longitudinal), residual confounding in unmatched designs, and non–trimester-specific observing could potentially account for these differences. We also report that an elevated ICH risk was observed in the first 12 weeks of the postpartum period, rather than the traditional 6-week postpartum period. To our knowledge, ICH risk during an extended postpartum period has not been evaluated previously, even though there are prior reports^6^ of an elevated risk of thromboembolic events beyond the conventional postpartum time period. These findings support the updated guidelines for postpartum care to be considered a care continuum, rather than a single postpartum visit at 6 weeks, similar to monitoring recommendations for peripartum cardiomyopathy.^17^ For patients with elevated risk profiles, such as those with hypertension or preeclampsia, the need for close, continued follow-up post partum should be emphasized.

In our cohort, approximately one-third (35.29%) of women who experienced ICH had eclampsia or preeclampsia. We also reported that ICH during pregnancy or the postpartum period was independently associated with hypertension, including gestational hypertension, coagulopathy, and history of tobacco use. Eclampsia and other related clinical states tend to produce hypertensive encephalopathy-like cerebrovascular changes, resulting in states of cerebral hyperperfusion and edema.^18^ Eclampsia is an important cause of serious and often fatal ICH, and our data corroborate those of prior reports,^5,16^ where ICH during pregnancy or the postpartum period has been commonly ascribed to eclampsia-related causes, coagulopathies, and other vascular pathologic abnormalities. Pregnancy is generally described as a state of hypercoagulation, which is thought to be an adaptive physiological response to reduce hemorrhage risk. However, systematic activation of the coagulation cascade by circulating thromboplastic factors followed by depletion of procoagulants and secondary activation of the fibrinolytic system results in disseminated intravascular coagulation. Disseminated intravascular coagulation manifests as life-threatening hemorrhage and is a major contributor to maternal mortality.^19^ In our data, 9.20% of patients with ICH also had a diagnosis of nonspecific coagulopathy, among whom one-half had disseminated intravascular coagulation. These factors were independently associated with ICH risk during pregnancy. It is possible that nonspecific coagulopathies in coding of administrative data also include other rare yet life-threatening complications of pregnancy and eclampsia, such as HELLP (hemolysis, elevated liver enzymes, and low platelet count) syndrome.^20^ Thrombocytopenia, although significantly associated with ICH risk in univariable analysis, was not independently associated with ICH risk in our adjusted model.

We found an increased independent risk of ICH among women of nonwhite race. This finding is consistent with previously reported higher rates of ICH in pregnancy for black women compared with white women.^7,16^ In addition, in our adjusted models, Hispanic and Asian women were also more likely to experience ICH during pregnancy compared with white women. Although this has not been demonstrated previously, our findings could be the result of a larger representation of Hispanic and Asian women in our study population compared with prior reports. Our findings are also consistent with overall maternal mortality and morbidity trends seen in the US.^19^ It is possible that our analyses reflect more recent trends in nationwide disparities in maternal mortality of all nonwhite patients compared with white patients.^7,16,19^ The drivers of racial disparities in maternal morbidity and mortality are most likely multifactorial.^19^ We also report a higher risk of ICH in pregnancy and the postpartum period with advanced maternal age and with tobacco use. Higher risk of pregnancy-related ICH with advanced maternal age is consistent with prior reports,^16,21^ and may be attributable to higher prevalence of risk factors, including hypertension and oral anticoagulation use among older women.^22^ With more women having children later in life,^23,24^ this association could account for the previously reported increased rate of stroke, including hemorrhagic stroke, during pregnancy and the postpartum period.^25^ Similarly, the association of pregnancy-related ICH with tobacco use is also consistent with prior reports,^7,16^ and with increased ICH risk associated with tobacco use in the general population.^26^

Our data indicate that women with ICH during pregnancy and the postpartum period are at high risk of maternal mortality. Specifically, women with ICH during pregnancy and the postpartum period were approximately 85 times more likely to die compared with women without ICH during delivery or within 52 weeks after delivery (adjusted OR, 84.69; 95% CI, 53.59-133.84). We also report an association between maternal ICH and fetal death (unadjusted OR, 5.40; 95% CI, 2.67-10.92). Surviving offspring may also be at a higher risk of long-term cardiovascular risk factors,^27^ stroke,^28^ and autism^29^ among preeclampsia- or eclampsia-associated ICH cases. Regardless, remarkably high fatality rates associated with pregnancy-related ICH and poor fetal outcomes remain a major obstetric and public health concern. Given the lack of specific ICH treatment modalities, early identification and management of risk factors and close monitoring during pregnancy and the extended postpartum period continue to be vitally important.

We also evaluated other potential causes of ICH during pregnancy and the postpartum period in our data. The influence of physiological changes associated with pregnancy and the postpartum period on rupture rates of arteriovenous malformations remains controversial. In both the cohort and crossover periods, the number of patients with ICH and arteriovenous malformation was fewer than 10. This is lower than numbers in prior reports using the same *ICD-9* code and could be the result of inclusion of all intracranial hemorrhages (ie, subarachnoid hemorrhage) rather than solely ICH.^7,30,31^ Although it is possible that arteriovenous malformation was underdiagnosed, our data suggest that it is unlikely that arteriovenous malformation rupture significantly contributes to ICH incidence reported here. Similarly, we identified fewer than 10 patients with cerebral venous sinus thrombosis and no cases of sickle cell anemia in our analysis cohort.

### Limitations

Our results should be interpreted in light of certain limitations. First, the identification of cohort and comorbidities depends on diagnosis and procedural administrative codes and does not include information such as presentation characteristics. In analyses of administrative data, misclassification is possible because of coding errors or because of differences between coding practices at different hospitals and over time. Furthermore, we assumed a uniform gestation period of 40 weeks for all pregnancies that could have led to trimester misclassification. However, we used a validated *ICD-9* code for ICH that has shown to have a high degree of sensitivity, specificity, and positive predictive value.^8,9,10^ We also used previously reported *ICD-9* codes for the study of stroke in pregnancy and the postpartum period^6,7^ and restricted the time period of our analyses to avoid *ICD-9* to *International Statistical Classification of Diseases and Related Health Problems, Tenth Revision* transitions. Second, we used in-hospital databases, which exclude labor and delivery events at home, birthing centers, or other nonhospital environments. The potential effect of this exclusion may be minimal, however, because in 2012 only 1.4% of deliveries in the US occurred in a nonhospital setting.^32^ Third, women with a history of ICH may be less likely to become pregnant and women may have relocated out of state during the follow-up period. To address this potential limitation, we conducted 2 sensitivity analyses. In the first, women were observed for ICH 64 weeks before pregnancy, which also resulted in a significantly increased risk of ICH during the third trimester (RR, 8.00; 95% CI, 4.00-16.00) and the first 12 weeks of the postpartum period (RR, 16.00; 95% CI, 7.46-34.34). In the second analysis, we excluded women without a hospital admission or emergency department visit after the 52-week interim period to account for potentiality of relocating out of state. This analysis continued to demonstrate an elevated ICH risk during the extended (12-week) postpartum (RR, 4.38; 95% CI, 2.40-8.01). Furthermore, although we followed patients longitudinally, only diagnoses in the inpatient and emergency department environment were included in analysis. This could result in an underestimate of certain comorbidities and risk factors, such as blood pressure management and social determinants of health. However, the cohort-crossover design minimizes the effect of unmeasured confounding.

## Conclusions

These findings provide a robust population-level estimate of risk of ICH during pregnancy and show that an elevated ICH risk continues into the extended 12-week postpartum period. Intracerebral hemorrhage during pregnancy is associated with hypertension, eclampsia, and coagulopathies, and older nonwhite women may be at a higher risk of ICH during pregnancy. Pregnancy-related ICH has an extremely poor maternal and fetal prognosis, and, as reported previously,^7^ ICH continues to be one of the significant drivers of pregnancy-related maternal mortality in the US. To reduce the burden of maternal and fetal mortality and morbidity due to ICH, there is a continued need for identification of high-risk pregnancies and proactive monitoring and management of ICH-associated risk factors, particularly during the later stages of pregnancy into the extended postpartum period.
